# 23-gauge transconjunctival vitrectomy in eyes with pre-existing functioning filtering blebs

**DOI:** 10.1186/s12886-015-0069-0

**Published:** 2015-07-24

**Authors:** Seong Taeck Kim, Gwang Rae Shin, Ji Man Park

**Affiliations:** Department of Ophthalmology, Chosun University School of Medicine, 365# Philmun-daero street, Dong-gu, Gwangju District 501-717 Republic of Korea

**Keywords:** Trabeculectomy, Filtering bleb, 23-gauge transconjunctival vitrectomy

## Abstract

**Background:**

We investigated the outcome of 23-gauge transconjunctival pars plana vitrectomy (23G PPV) for the treatment of vitreoretinal disorder in patients with prior trabeculectomy.

**Methods:**

We retrospectively reviewed medical records of 23G PPV in 11 eyes that had functioning filtering blebs after trabeculectomy. The main outcome measures were the visual acuity, intraocular pressure (IOP) and subconjunctival fluid height in bleb by anterior segment optical coherence tomography (OCT) before and after the surgery.

**Results:**

Eyes that had 23G PPV showed improvement of visual acuity after the surgery (*P* =0.003). Mean IOP was 13.82 mmHg before 23G PPV and 15.82 mmHg at 6 months postoperatively, which was statistically insignificant (*P* = 0.758). The bleb was observed before and after surgery using anterior segment OCT, and the difference in subconjunctival fluid was not statistically significant (*P* =0.172).

**Conclusions:**

23G PPV did not adversely affect bleb function in eyes with prior trabeculectomy.

## Background

Trabeculectomy has become the most widely performed and successful surgical method for glaucoma since it was first presented by Cairns in 1968 [1]. Trabeculectomy drains the aqueous humour in eye into the subconjunctival space, which subsequently spreads out from the subconjunctiva to adjust intraocular pressure (IOP). Morphology of the bleb is closely related to success of the trabeculectomy [2]. Occasionally, patients with functioning filtering blebs may require pars plana vitrectomy (PPV) for various vitreoretinal disease. Although several studies have evaluated the effect of cataract extraction on functioning filtering bleb trabeculectomy outcome, only a few have focused on the impact of PPV on filtering bleb function [3]. Thompson et al. reported that it is difficult to maintain bleb function when 20-gauge (20G) conventional vitrectomy is performed after trabeculectomy [4].

Vitreoretinal surgery has recently been developed as a minimal invasive technique. Transconjunctival sutureless vitrectomy induces to simplify the surgical process, reduce postsurgical inflammation, and promote rapid patient recovery [5]. It can protect and preserve conjunctiva that can be damaged by standard 3-port 20G PPV and shorten the wound healing period [6]. Eyes with prior trabeculectomy would require microincision vitrectomy for preservation of the filtering bleb. Kunikata et al. recently reported the good outcomes of 25-gauge (25G) microincision PPV in eyes that received prior trabeculectomy [7]. However, there are no such studies on outcome of 23-gauge (23G) PPV in eyes that received prior trabeculectomy. Moreover, there are few reports on researches of the morphological changes of bleb by anterior segment optical coherence tomography (OCT) after PPV. Therefore, in the current study, we evaluated the outcome of functioning filtering blebs in eyes undergoing 23G PPV.

## Methods

### Patients

Among patients who received trabeculectomy for lowering IOP at Chosun University Hospital from January 2008 to April 2014 and who maintained filtering bleb function, 11 eyes with ≥ 6 months follow-up after 23G PPV for vitreoretinal disease were retrospectively analyzed based on medical records. The clinical and surgical records of these patients were evaluated for the following preoperative eligibility criteria: 1) trabeculectomy performed prior to 23G PPV, 2) elevated trabeculectomy bleb (not fibrotic and flattened), and 3) IOP between 6 and 20 mmHg. All retinal diseases that required 23G PPV were included. Cases with the 20G instrument mixed during surgery, prior scleral buckling or encircling, a follow-up period of < 6 months were excluded. All retinal surgeries were performed by a single surgeon at our hospital. This retrospective study was approved by the Institutional Review Board of Chosun University Hospital, Gwangju City, Republic of Korea. All procedures conformed to the Declaration of Helsinki. All participants gave informed consent after a detailed explanation of the study design, ancillary investigations for scientific purposes, and related imaging procedures.

### Surgical technique

All surgeries were implemented by retrobulbar anesthesia. 23G PPV used the 2-step 23G surgical method and instrument, as well as Millennium vitrectomy system^™^ (Bausch & Lomb, St. Louis, MO, USA) and Stellaris vitrectomy system^™^ (Bausch & Lomb, Rochester, NY, USA). The conjunctiva was pushed and fixed by a pressure plate, and sclerotomy was performed 3.0 ~ 3.5 mm from the limbus before inserting the cannula. After inserting the infusion cannula into the inferotemporal sclera, remaining 2 cannulas were inserted into the superotemporal and superonasal sites. There was no surgical protocol for preserving the filtering bleb, but the vitreoretinal surgeon in general avoided disturbing the conjunctiva adjacent to the filtering bleb by shifting the meridians of sclerotomy as necessary. In one case, the bleb was extended to 270°, and the surface of the bleb was pressed by a pressure plate followed by insertion of the cannula through the bleb. In this case, conjunctival suture was performed to prevent bleb leakage after 23G PPV. When cataract surgery was necessary, 2.2 mm superior clear corneal incision and phacoemulsification and intraocular lens (IOL) implantation were carried out after inserting the cannula and blocking with a plug. The entire surgical procedure was carried out based on each disease. 4 mg of triamcinolone acetonide (Triam®, Shinpoong, Korea) was injected into the vitreous cavity to determine if a posterior vitreous detachment was present. If a posterior vitreous detachment was not present, we created one with a vitrectomy cutter or soft-tipped extrusion cannula. Severe conjunctival chemosis interfered with the operative proceedings in one case. We accordingly applied a small incision on the conjunctiva and Tenon’s capsule to drain the fluid and sutured the conjunctiva after the operation. If it was thought that the sclerotomy site would not self-seal due to leakage, the sclerotomy site was closed with 8–0 Vicryl® (Polyglactin 910, Ethicon, Inc., USA) sutures. As sclerotomy site was invisible, sclerotomy suture was performed after conjunctival incision and conjunctival suture was performed layer by layer. Postoperatively, only antibiotics were injected subconjunctivally, avoiding the filtering bleb. Topical treatment, administering the fluoroquinolone (Cravit; Santen, Osaka, Japan) four times per day for 1 month and a 1 % prednisolone acetate (Pred-forte; Allergan, Irvine, CA, USA) was administered four times daily for the two weeks and tapered over 2 months before being discontinued.

### Surgical outcome measures and observation procedure

Sex and age of all patients were obtained from medical records. Diagnosis of glaucoma for which trabeculectomy was performed, method of conjunctival flap, position of bleb, and use of mitomycin C (MMC) or 5-fluorouracil (5-FU) were recorded. Diagnosis of retinal disease for which 23G PPV was implemented, combined cataract surgery in 23G PPV, status of sclerotomy port suturing, and status of subconjunctival hemorrhage were evaluated. Period between trabeculectomy and 23G PPV and follow-up period after 23G PPV surgery were also determined. For change in best-corrected visual acuity (BCVA) according to 23G PPV, preoperative BCVA and BCVA measured at least 6 months after the surgery were compared. They were converted into logarithm of the minimal angle of resolution (LogMAR) units for statistical analysis. For evaluation of visual acuity, “count fingers” was regarded as 2.0 logMAR units and “hand motion” as 3.0 log-MAR units. Comparative change in IOP was determined preoperatively and at 1 day, 1 week, 1 month, 3 months and 6 months postoperatively. The status of additional topical glaucoma medication was also investigated.

**Fig. 1:**
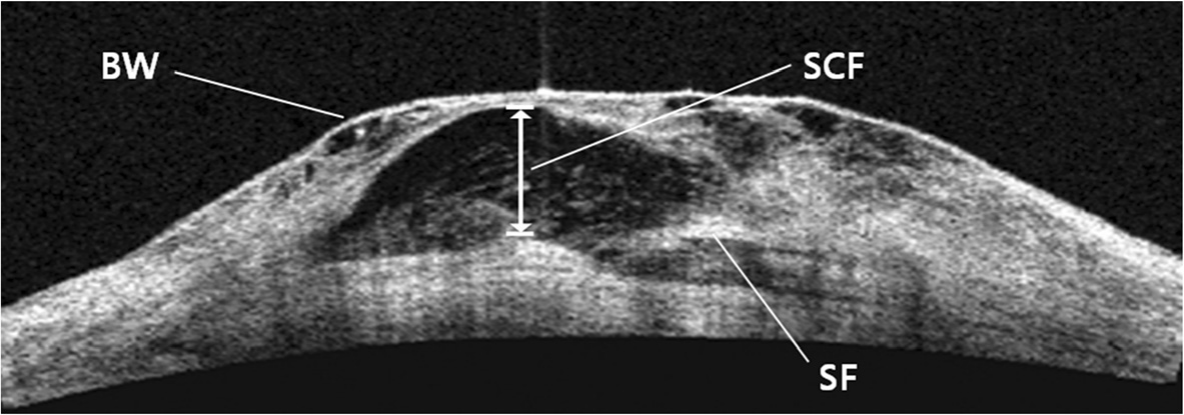
Morphology of bleb by Visante^TM^ OCT. BW = bleb wall; SCF = subconjunctival fluid; SF = scleral flap

The subjects’ internal bleb structure was visualized by anterior segment OCT with the Visante^™^ OCT Model 1000 (Anterior segment optical coherence tomography: Carl Zeiss Meditec). Upon selection of the examination protocol, ‘bleb’ mode was used to perform the scanning. The bleb was vertically scanned several times starting from the highest point, and analysis was done on the scan that most accurately showed the internal structure. Subconjunctival fluid was somewhat irregular because of microcyst or cyst located under the bleb wall on Visante^™^ OCT, and was defined as the space with low to medium shade (Fig. 1). Height of subconjunctival fluid was measured and compared preoperatively, 1 week, 1 month, and 6 months post-surgery.

### Statistical analysis

Statistical analysis was performed using SPSS® version 17.0 for Windows software (SPSS, Chicago, IL, USA). All data were displayed as mean ± standard deviation. The significance of difference in the preoperative BCVA and the final BCVA was determined by the Wilcoxon signed rank test. Significant differences in IOP before and after 23G PPV were determined by the Friedman test. Significant differences in subconjunctival fluid of bleb before and after 23G PPV were determined by the Friedman test. The level of statistical significance was determined as *P*-value < 0.05.

## Results

The clinical characteristics of the 11 eyes of 11 patients with functioning filtering blebs who underwent 23G PPV were listed in Tables (Tables 1 and 2). Mean age of all subjects was 64.3 ± 7.1 years, and 7 subjects were males and 4 were females. The most common type of glaucoma that received trabeculectomy was open angle glaucoma in 4 eyes (36 %), followed by neovascular glaucoma in 3 eyes (27 %), primary angle closure glaucoma in 1 eye (9 %), traumatic glaucoma in 1 eye (9 %), uveitis-associated secondary glaucoma in 1 eye (9 %), and pseudoexfoliation glaucoma in 1 eye (9 %). A limbus-based conjunctival flap method was used in 1 case, and fornix-based conjunctival flap was used in the rest of the cases. Most of the blebs were located in the upper nasal quadrants; however, in 1 case the bleb was located in the upper temporal quadrant in because conjunctival suture had ever been performed at the upper nasal quadrant for traumatic laceration. MMC was used during trabeculectomy in all cases, and 5-FU was used in 2 eyes (18 %). Mean time between trabeculectomy and 23G PPV was 25.3 ± 29.7 months (A range of 3 to 47 months).

**Table 1:** Characteristics of patients with pre-23-gauge vitrectomy course of 11 glaucomatous eyes

Patient	Sex/Age	Diagnosis of glaucoma	Conjunctival flap	Site of bleb	MMC	5-FU	TLE ~ PPV (months)
1	M/63	NVG	Fornix based	Upper nasal	O	O	15
2	F/71	NVG	Fornix based	Upper nasal	O	X	41
3	M/55	NVG	Fornix based	Upper nasal	O	X	33
4	M/53	POAG	Fornix based	Upper nasal	O	X	11
5	M/72	POAG	Fornix based	Upper nasal	O	X	47
6	F/73	POAG	Fornix based	Upper nasal	O	X	39
7	F/57	POAG	Fornix based	Upper nasal	O	X	14
8	F/69	PACG	Fornix based	Upper nasal	O	X	7
9	M/60	Trauma	Fornix based	Upper temporal	O	X	3
10	M/68	Uveitis	Limbal based	Upper nasal	O	O	31
11	M/66	PXF	Fornix based	Upper nasal	O	X	37

*FU* fluorouracil, *MMC* mitomycin C, *NVG* neovascular glaucoma, *PACG* primary angle closure glaucoma, *POAG* primary open angle glaucoma, *PPV* pars plana vitrectomy, *PXF* pseudoexfoliation syndrome, *TLE* trabeculectomy

**Table 2:** Characteristics of patients with post-23-gauge vitrectomy course of 11 post-trabeculectomy eyes

Patient	Sex/Age	Diagnosis for PPV	Combined cataract surgery	Port suturing	SCH	Baseline BCVA	Final BCVA	Followup (months)
1	M/63	PDR	X	X	O	HM	0.05	15
2	F/71	PDR	X	X	X	0.01	0.2	23
3	M/55	PDR	X	X	X	HM	0.1	13
4	M/53	ERM	O	X	X	0.1	0.5	12
5	M/72	ERM	X	X	O	0.2	0.4	6
6	F/73	MH	X	O	X	0.02	0.2	19
7	F/57	VH d/t BRVO	X	X	X	HM	0.5	22
8	F/69	Lens dislocation	O	X	O	0.05	0.6	27
9	M/60	RRD	O	X	X	0.02	0.4	24
10	M/68	ERM	O	X	X	0.2	0.5	7
11	M/66	ERM	X	X	X	0.15	0.4	9

*BCVA* best corrected visual acuity, *BRVO* branch retinal vein occlusion, *d/t* due to, *ERM* epiretinal membrane, *HM* hand motion, *MH* macular hole, *PDR* proliferative diabetic retinopathy, *PPV* pars plana vitrectomy, *RRD* rhegmatogenous retinal detachment, *SCH* subconjunctival hemorrhage, *VH* vitreous hemorrhage

The most common type of vitreoretinal disease that required 23G PPV was epiretinal membrane in 4 eyes (36 %), followed by proliferative diabetic retinopathy in 3 eyes (27 %), rhegmatogenous retinal detachment in 1 eye (9 %), macular hole in 1 eye (9 %), vitreous hemorrhage due to branch retinal vein occlusion in 1 eye (9 %), and dislocated intraocular lens in 1 eye (9 %). Simultaneous surgery of cataract and 23G PPV was performed in 4 eyes (36 %). None of the eyes was required suturing of the sclerotomy site at the end of the surgery except one (9 %) that had undergone gas tamponade. The conjunctival suture was performed to prevent bleb leakage in the case of severe conjunctival chemosis and extensive bleb. While subconjunctival hemorrhage occurred in 3 eyes (27 %), none invaded the filtering bleb. Mean preoperative BCVA (logMAR) was 1.8 ± 0.9 and mean final BCVA was 0.6 ± 0.5. These values were statistically different (*P* =0.003).

**Fig. 2:**
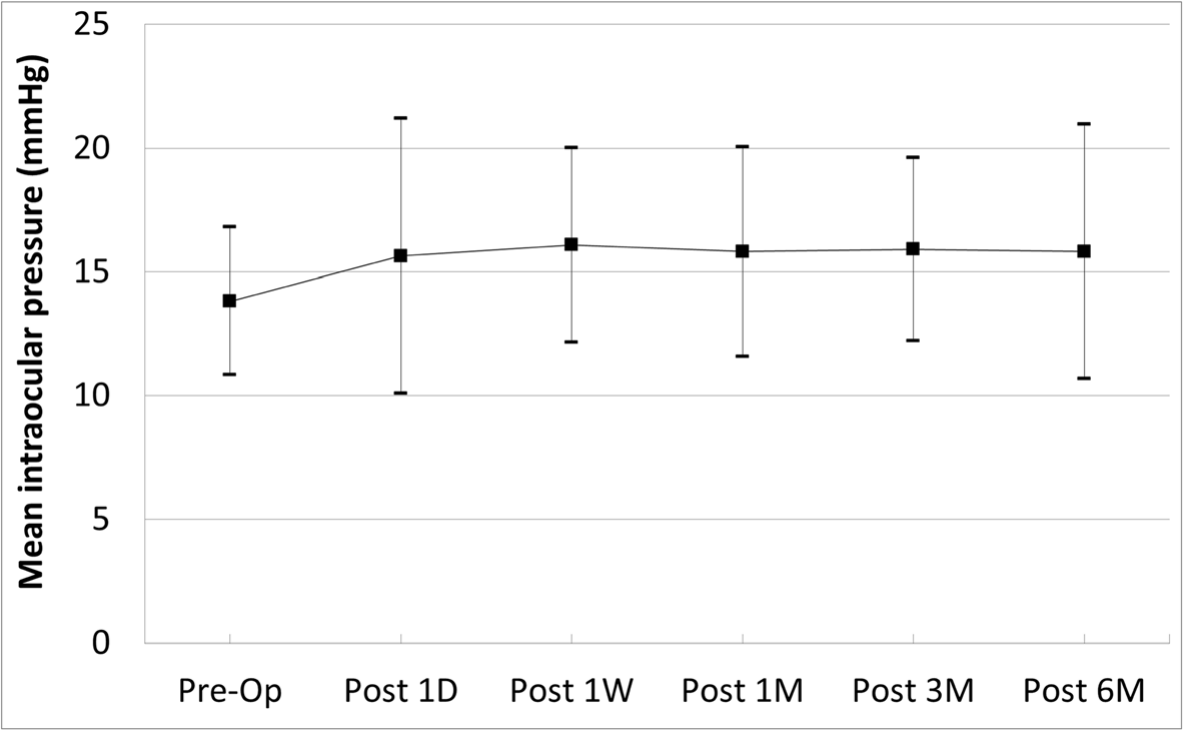
Changes of the intraocular pressure (IOP) after 23-gauge vitrectomy in eyes with functioning filtering blebs. The difference in IOP was statistically insignificant (*P* =0.758). Op = operation; D = day; W = week; M = month

Mean IOP was 13.82 ± 2.99 mmHg before 23G PPV, 15.64 ± 5.56 mmHg at 1 day after the surgery, 16.09 ± 3.93 mmHg at 1 week after the surgery, 15.82 ± 4.24 mmHg at 1 month after the surgery, 15.91 ± 3.70 mmHg at 3 months after the surgery and 15.82 ± 5.14 mmHg at 6 months after the surgery. The differences were not statistically significant (*P* = 0.758) (Fig. 2). Median IOP was was 14 mmHg before 23G PPV, 14 mmHg at 1 day after the surgery, 16 mmHg at 1 week after the surgery, 16 mmHg at 1 month after the surgery, 16 mmHg at 3 months after the surgery and 15 mmHg at 6 months after the surgery. Three eyes (27 %) had intraocular pressure > 21 mmHg at the 1 day after 23G PPV, and 1 of these eyes had IOP persistently greater than 21 mmHg. Our study showed that there was no hypotony (IOP < 5 mmHg) after 23G PPV. The early postoperative IOP was increased in the case with gas tamponade, but was controlled within 2 weeks. Topical glaucoma medication was used in 4 eyes before the surgery. The number of topical glaucoma medication was increased from preoperative to the final follow-up in 2 eyes. Among them, IOP was not controlled by topical glaucoma medication in 1 eye (proliferative diabetic retinopathy patient), requiring additional glaucoma surgery.

**Fig. 3:**
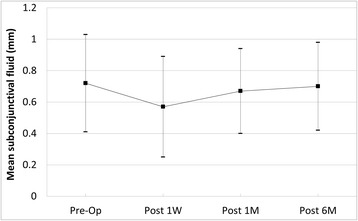
Changes of the subconjunctival fluid after 23-gauge vitrectomy in eyes with functioning filtering blebs. Height of subconjunctival fluid was measured by anterior segment optical coherence tomography (OCT) and compared. The difference in subconjunctival fluid was statistically insignificant (*P* =0.172). Op = operation; D = day; W = week; M = month

Filtering bleb was observed in all 11 eyes on slit lamp microscopy before 23G PPV. Temporary loss of bleb occurred in 2 eyes (18 %) after 23G PPV, but they were reformed in 2 ~ 4 weeks after the surgery. Bleb before and after the surgery was observed using anterior segment OCT, and the difference in subconjunctival fluid between groups was not significant (*P* =0.172) (Fig. 3). Mean length of follow-up after the surgery was 16.1 ± 7.3 months (range of 6 to 27 months). Height of subconjunctival fluid was measured by anterior segment optical coherence tomography (OCT) and compared.

## Discussion

Trabeculectomy and vitrectomy are procedures most often performed in glaucoma and retinal disease. The number of patients with functioning filtering bleb may require PPV for various vitreoretinal disease. Eyes that receive prior trabeculectomy can lead to problems if further retinal diseases requiring vitrectomy. Trabeculectomy has long been selected as an effective surgical method to maintain low IOP for prolonged periods of time. Antimetabolites such as MMC or 5-FU can be used to increase the success rate of surgery. MMC or 5-FU regulates the wound healing process of bleb by suppressing subconjunctival fibrosis that is the major cause of surgical failure and also affects bleb function and morphology [8]. Preservation of conjunctiva after the surgery is important because success of the surgery depends on the condition of bleb after trabeculectomy. Thus, when patients with functioning filtering bleb may be required PPV, it is considerable factor how well the filtering bleb is preserved. In 1976, Kolker and Heathering reported a 50 % loss of filtering bleb in intracapsular cataract extraction carried out after trabeculectomy [9]. However, with development of phacoemulsification, Alpar reported that loss of filtering bleb occurred in only 1 of 7 eyes with phacoemulsification after trabeculectomy [3]. Likewise, when vitrectomy is needed in eyes that receive prior trabeculectomy, it would be more advantageous to use transconjunctival sutureless microincisional PPV than the 20G conventional PPV. Thompson et al. reported that it is difficult to maintain bleb function when 20G conventional PPV is performed after trabeculectomy [4]. Vitreoretinal surgery has recently been developing as minimal invasive technique. Eyes with prior trabeculectomy require microincision vitrectomy for preservation of the filtering bleb. In addition, conjunctival scarring occurs in most cases after 20G conventional PPV, and transconjunctival sutureless microincisional vitrectomy can minimize conjunctival scarring and increase success rate of trabeculectomy later on [10]. There was a case in 2007 that reported successful outcome of 25G PPV in familial amyloid polyneuropathy patients with filtering bleb [11]. Kunikata et al. recently reported the stable results of 25G microincision vitrectomy in eyes that received trabeculectomy [7]. Therefore, the authors evaluated 23G PPV for vitreoretinal disease in eyes with prior trabeculectomy.

In this study, 36 % of the subjects had open angle glaucoma for which trabeculectomy was performed, followed by 27 % with neovascular glaucoma. Kunikata et al. reported 53 % neovascular glaucoma and 13 % open angle glaucoma cases, and Thompson et al., reported 57 % open angle glaucoma and 17 % angle closure glaucoma [4, 7]. In the current study, 4 eyes (36 %) of the 11 subjects had epiretinal membrane and 3 eyes (27 %) of the 11 subjects had proliferative diabetic retinopathy. According to Kunikata et al., 53 % of patients who received 25G PPV in eyes that received trabeculectomy were proliferative diabetic retinopathy patients, and only 1 eye (6.5 %) was from an epiretinal membrane patient [7]. Thompson et al., studied patients with 20G PPV in eyes that received trabeculectomy, of which 57 % had rhegmatogenous retinal detachment and 13 % each had proliferative diabetic retinopathy and epiretinal membrane [4]. The high ratio of epiretinal membrane in our study is likely due to the expansion of epiretinal membrane surgery indication by the development of OCT compared to before. Thompson et al. reported that the use of prior antimetabolite was not correlated with preservation of bleb function [4]. We had statistical difficulty comparing the use and non-use of prior antimetabolite because all cases used antimetabolite. Although the use of prior antimetabolite does not affect preservation of bleb function, measures such as 5-FU injection after PPV are believed to maintain bleb function. Nonetheless, since the possibility of bleb failure is low in transconjunctival sutureless microincisional vitrectomy as in 23G PPV, 5-FU injection can be attempted immediately after PPV when bleb failure is a risk.

BCVA was improved in all cases after 23G PPV, which implies that the status of bleb does not affect the success rate of vitrectomy. Thompson et al., reported increased IOP in about 1/3 of vitrectomy performed in eyes that received trabeculectomy, which was corroborated by our study [4]. In our study, IOP was increased after the surgery, as compared to mean IOP before 23G PPV, but the difference not statistically significant. In the case of gas tamponade, the early postoperative IOP was increased but controlled under topical glaucoma medication within 2 weeks. Topical glaucoma medication was used in 4 eyes before the surgery. The number of topical glaucoma medication was increased between preoperative and final follow-up in 2 eyes. Among them, IOP was not adjusted by topical glaucoma medication in 1 eye (proliferative diabetic retinopathy patient) that required additional glaucoma surgery, after which IOP was stabilized. Kunikata et al. reported that the IOP increased to ≥ 20 mmHg in 30 % of subjects after 25G PPV, of which 3 eyes required additional glaucoma surgery. All cases were neovascular glaucoma patients caused by proliferative diabetic retinopathy [7]. Conjunctival scarring occurs in most cases after 20G conventional PPV, but transconjunctival sutureless microincisional PPV minimizes conjunctival scarring to increase the success rate of surgery without special surgical difficulty in additional trabeculectomy. While Thompson et al. reported increased IOP in 1/3 subjects after 20G PPV and occurrence of hypotony in 1/3, we found no case of hypotony with IOP of ≤ 5 mmHg after 23G PPV, similar to the results of Kunikata et al. [4, 7]. The inter-study differences probably resulted from relatively more hypotonic eyes with persistent retinal detachments after vitrectomy reported by Thompson et al. [4].

Thompson et al. reported that bleb function was only maintained in 1/3 of cases where vitrectomy was performed after trabeculectomy [4]. In trabeculectomy, the morphology of bleb was closely related to the success of the surgery. However, there are few reports on researches of the morphological changes of bleb after PPV. Various classification methods were introduced to evaluate the morphology of bleb, and representative methods include classification method by Kronfeld, Indiana bleb appearance grading scale (IBAGS), and Moorfields bleb grading system scale (MBGS) [12–14]. However, such classification methods are based on subjective opinions obtained from slit lamp examination that are not quantitative and cannot reflect the internal structure of the bleb. Ultrasound biomicroscopy (UBM) was the first method introduced to observe internal morphology of the bleb [15]. However, UBM is limited in that it requires contact and axial resolution can reach only 25 μm [16]. On the contrary, anterior segment OCT widely used for clinical purpose is a non-contact and non-invasive method with high resolution of 10 μm. It is the most accurate and easy method for estimating internal structure and function of bleb [17]. Therefore, to evaluate bleb in this study, the hyporeflective space on anterior segment OCT was defined as subconjunctival fluid to compare height. Existence of subconjunctival fluid in the internal structure image indirectly suggests continuous leakage of aqueous humour, and height of fluid in the subconjunctival space can be used as an important factor of bleb function [18]. The height of subconjunctival fluid compared between preoperative, 1 week, 1 month, and 6 months after the surgery in this study showed no statistically significant differences. Transient loss of filtering bleb occurred in 2 eyes (18 %) postoperatively, but it was reformed in 2 ~ 4 weeks. Loss of bleb function may occur temporarily in the early postoperative period. Extensive bleb extending 270° was found in 1 case, and the cannula was inserted through the bleb and conjunctival suture was performed to prevent bleb leakage. There was no difference in IOP before and after the surgery. Conjunctival edema that occurs during surgery negatively affects bleb function by invading the bleb margin; however, we found no differences in IOP before and after the surgery. Therefore, when 23G PPV is done in the trabeculectomized eye, it is helpful to use a pressure plate and adhere space between the conjunctiva and sclera to prevent conjunctival edema. Since there was no significant difference in IOP caused by conjunctival edema, as indicated by the study results, the leaking status was checked to perform conjunctival suture when necessary.

Conventional 20G PPV can be accompanied by injuries such as episcleral and conjunctival vessel, thus affecting filtering bleb function. Hemorrhage can affect the pre-existing filtering bleb. Based on this principle, many existing studies reported autologous blood injection for treatment of overfiltering or leaking blebs after glaucoma surgery [19, 20]. Thus in case of sutureless 23G PPV, subconjunctival hemorrhage is minimized during the surgery to minimize the effect on bleb. Subconjunctival hemorrhage occurred in 27 % of the study subjects, which was similar to the occurrence of subconjunctival hemorrhage in 30 % of cases observed by Kunikata et al. [7]. Care is required to prevent damage to conjunctival vessel during 23G PPV, especially during sclerotomy in eyes that received trabeculectomy. When bleeding occurs at the sclerotomy site, bleeding must be minimized by positioning of epinephrine gauze. Gotzardis reported that diathermy induces temporary adhesion between conjunctiva and sclera, which could reduce the risk of subconjunctival hemorrhage and conjunctival chemosis [21].

The current study had some limitations. First, retrospective analysis commonly has more sources of error due to confounding factors and bias. Second, the sample size was too small because it is difficult to collect a large number of such cases. Better results can be expected by gathering more cases and comparing with the group that received 23G PPV in eyes without history of glaucoma surgery. In addition, we did not compare 23G PPV and 20G PPV.

## Conclusions

We examined the effect of 23G PPV on IOP and bleb in eyes that received 23G PPV after trabeculectomy. Eyes that received 23G PPV showed significant improvement of visual acuity and stable IOP. Bleb was observed before and after the surgery using anterior segment OCT, and there was no significant difference in subconjunctival fluid between groups. The results of this study suggested that 23G PPV did not adversely affect bleb function in eyes with prior trabeculectomy.

## Abbreviations

PPV: Pars plana vitrectomy
IOP: Intraocular pressure
OCT: Optical coherence tomography
BCVA: Best-corrected visual acuity
LogMAR: Logarithm of the minimal angle of resolution
FU: Fluorouracil
MMC: Mitomycin C
NVG: Neovascular glaucoma
PACG: Primary angle closure glaucoma
POAG: Primary open angle glaucoma
PXF: Pseudoexfoliation syndrome
TLE: Trabeculectomy
BRVO: Branch retinal vein occlusion
ERM: Epiretinal membrane
HM: Hand motion
MH: Macular hole
PDR: Proliferative diabetic retinopathy
RRD: Rhegmatogenous retinal detachment
SCH: Subconjunctival hemorrhage
VH: Vitreous hemorrhage
